# A well-trained artificial neural network for predicting the rheological behavior of MWCNT–Al_2_O_3_ (30–70%)/oil SAE40 hybrid nanofluid

**DOI:** 10.1038/s41598-021-96808-4

**Published:** 2021-08-31

**Authors:** Mohammad Hemmat Esfe, S. Ali Eftekhari, Maboud Hekmatifar, Davood Toghraie

**Affiliations:** 1 Department of Mechanical Engineering, Imam Hossein University, Tehran, Iran; 2 Department of Mechanical Engineering, Khomeinishahr Branch, Islamic Azad University, Khomeinishahr, Iran

**Keywords:** Engineering, Nanoscience and technology

## Abstract

In this study, the influence of different volume fractions ($\phi$) of nanoparticles and temperatures on the dynamic viscosity ($\mu_{nf}$) of MWCNT–Al_2_O_3_ (30–70%)/oil SAE40 hybrid nanofluid was examined by ANN. For this reason, the $\mu_{nf}$ was derived for 203 various experiments through a series of experimental tests, including a combination of 7 different $\phi$, 6 various temperatures, and 5 shear rates. These data were then used to train an artificial neural network (ANN) to generalize results in the predefined ranges for two input parameters. For this reason, a feed-forward perceptron ANN with two inputs (T and $\phi$) and one output ($\mu_{nf}$) was used. The best topology of the ANN was determined by trial and error, and a two-layer with 10 neurons in the hidden layer with the tansig function had the best performance. A well-trained ANN is created using the *trainbr* algorithm and showed an MSE value of 4.3e−3 along 0.999 as a correlation coefficient for predicting $\mu_{nf}$. The results show that an increase $\phi$ has a significant effect on $\mu_{nf}$ value. As $\phi$ increases, the viscosity of this nanofluid increases at all temperatures. On the other hand, with increasing temperature, the viscosity of this nanofluid decreases. Based on all of the diagrams presented for the trained ANNs, we can conclude that a well-trained ANN can be used as an approximating function for predicting the $\mu_{nf}$.

## Introduction

Today, the issue of heat transfer in power plants and industries has become a major challenge^1–7^. Researchers are always looking to increase the efficiency of heating equipment with different methods^8–10^. Nanofluids are one of the newest and best ways to improve the thermal performance of fluid systems. Choi^11^ first coined the term nanofluid to describe very small particles (nanoparticles less than 100 nm in diameter) suspended in a fluid. In nanofluids, one or more solid particles are added to the fluid, which increases the rate of heat transfer and change in viscosity^12, 13^. Some nanoparticles are in the form of oxides and play an important role in the dispersion and suspension of fluid, and some are in the form of non-oxide metal particles^14–16^. One of the main properties of fluids is viscosity. Viscosity can be mentioned as the inhibitory force and the magnitude of the frictional properties of the fluid^17^. In addition, viscosity is a function of temperature and pressure^18^. Fluid viscosity is commonly used for engineering designs and the definition of dimensionless numbers such as Reynolds and Prandtl^19^. In addition, fluid viscosity is used to calculate the required power of pumps, mixing processes, piping systems, liquid pulverization, fluid storage, fluid injection, and fluid transport^20^. Hybrid nanofluids combine two heterogeneous nanoparticles (hybrid nanocomposites) suspended in the base fluid^21–24^. The purpose of using hybrid nanocomposites in an intermediate fluid is to improve the heat transfer characteristics of the base fluid through the combined thermophysical characteristics of effective nanomaterials^25–28^. In recent years, various fluids such as water, ethylene glycol, and various oils were used as operating fluids in industry and engineering design. Given the growing need for cooling systems with high heat losses due to viscosity changes, scientists and researchers have been encouraged to achieve fluids with higher heat transfer properties (increased heat transfer rate) and effective viscosity over temperature. The amount of viscosity in nanofluid design is very critical for fluid flow^29^. Due to pressure drops in the pump, fluid concentration is known to be important in industrial applications. In the last decade, researchers have presented various researches on thermophysical parameters (temperature, particle size φ, shape, size, impact of time or agglomeration, *μ*_*nf*_, and base fluids) and transverse theoretical and laboratory relations^30, 31^. The knowledge of predicting the test process is a powerful tool for engineers who want to design and produce their products with excellent quality and the lowest cost. Therefore, ANNs are considered computational methods in artificial intelligence systems and new computational methods to predict the output responses of complex systems^32–35^. The main idea of ​​such ANNs is, to some extent (inspired by how the biological neural system works to process data and information to learn and create knowledge. The key element of this idea is to create new structures for the information processing system. Many super-interconnected processing elements called neurons have formed to solve problems and transmit information through synapses (electromagnetic communication). ANNs are among the most advanced and modern methods in simulation^36–38^. Today, they have been widely used in all engineering sciences as a powerful tool in simulating phenomena whose conceptual analysis is difficult. In this method, the observational data is taught to the model, and after training the model, it performs forecasting and simulation work with appropriate accuracy. In recent years, researchers have used ANNs to predict the thermal conductivity of nanofluids and determine the appropriate *μ*_*nf*_^39–44^. For example, Miao et al.^45^ used ANNs to predict the *μ*_*nf*_ of a mixture of ethanol and methanol over a temperature range. The results show that the ANN model predicts the *μ*_*nf*_ of the compound with great accuracy. Yousefi et al.^46^ investigated the viscosities of metal oxides such as SiO_2_, Al_2_O_3_, CuO and TiO_2_ suspended in ethanol and water by the ANN method. The predicted results were in good agreement with the experimental results obtained. Therefore, this method is suitable for estimating the *μ*_*nf*_ of nanofluids containing metal oxide. Atashrouz et al.^47^ predicted the *μ*_*nf*_ of SiO_2_, Al_2_O_3_, CuO, and TiO_2_ nanofluids suspended on water, ethylene glycol, and propylene glycol by ANNs. The results show that this method is suitable for predicting the $\mu_{nf}$. Zhao et al.^48^ investigated the *μ*_*nf*_ of Al_2_O_3_ and CuO metal oxides water- suspended by ANNs. The predicted results were in good agreement with the experimental values obtained. Esfe et al.^49^ predicted the $\mu_{nf}$ of Fe/EG nanofluids by the ANN method. The predicted results are in good agreement with the experimental values obtained. Therefore, this method is very efficient in predicting the $\mu_{nf}$. Studies show that the prediction of $\mu_{nf}$ using ANNs is not very old and is being addressed by researchers. Therefore, this manuscript analyzed the influence of different φ alongside variable temperatures on μnf of MWCNT–Al_2_O_3_ (30–70%)/oil SAE40 hybrid nanofluid. For this reason, the $\mu_{nf}$ has been derived for 203 various experiments through a series of experimental tests, including a combination of 7 different $\phi$, 6 various temperatures, and 5 shear rates. These data were then used to train an ANN to generalize results in the predefined ranges for two input parameters. For this reason, a feed-forward Perceptron ANN with two inputs (T and $\phi$) and one output ($\mu_{nf}$) was used. The best topology of the ANN was determined by trial and error, and a two-layer with 10 neurons in the hidden layer with the tansig function had the best performance.

As observed in the literature, the use of post-processing methods such as artificial neural networks, response surface and optimization methods in various sciences, including nanofluid science has been very welcomed. The reason for this welcome is the reduction of time and financial costs in laboratory studies. However, the use of artificial neural networks and other post-processing methods to predict the behavior of nanofluids requires access to valid laboratory results. Hemmat Esfe Research Group is one of the active groups in the field of laboratory studies of nanofluids, which has provided more than 200 valuable experimental databases for other researchers to perform post-processing studies on various thermophysical properties of normal and hybrid nanofluids^50–56^. Feasibility studies of this research group in the field of application of nanofluids in increasing oil extraction^57,58^ and also the use of nanofluids in lubricants in order to minimize the damage caused by cold start of the car engine^59–61^ are other activities of this group.

## ANN configuration

**Figure 1:**
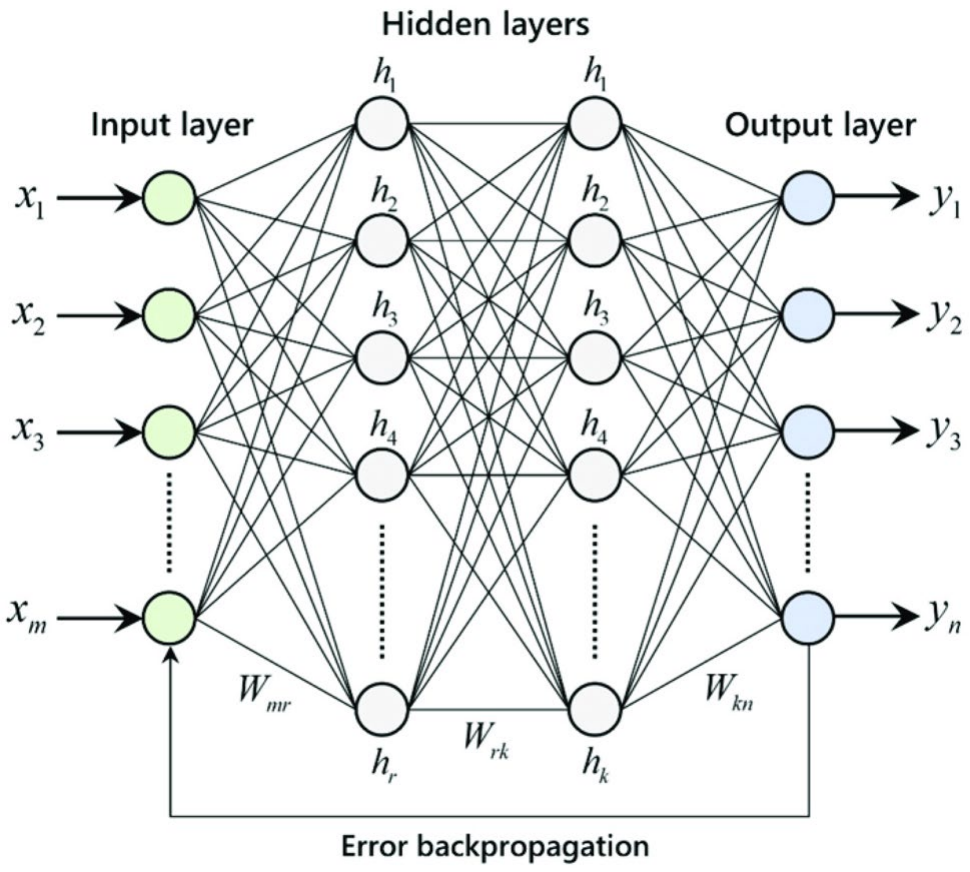
A multilayer perceptron ANN graph alongside inputs and outputs data.

An ANN is a powerful tool for processing raw data inspired by human brain structure and consists of many neurons that collaborate to model a system^62^. The configuration of ANNs is made up of several weighted elements. The nodes are the artificial neurons, and the directional arrows and the weights show the relationship between the outputs and the inputs of the neurons. ANNs are categorized into two groups based on their morphology: The first category is called feed-forward ANN, and the latter is called the recurrent ANNs. According to the experimental data in this study that are static and that feed-forward ANNs have high potential in function estimation, a feed-forward perceptron ANN has been used. The relationship between input and output data is nonlinear; therefore, a multilayer Perceptron ANN should simulate this nonlinear relationship. Accordingly, in this study, the network is constructed of two layers with nonlinear functions. This configuration has proven accuracy in function approximation in different studies. The backpropagation algorithms are efficient and effective; hence, several methods in this ANN training scheme are used, and their performance is compared. Different ANNs with various neuron numbers and transfer functions in the hidden layer have been examined. The best topology was determined by trial and error and minimizing the ANN error. The best results were obtained using 10 neurons in the second layer with hyperbolic tangent function and linear function in the output layer. A graph of a typical multilayer ANN is presented in Fig. 1 alongside different inputs and outputs.

For training an ANN, the first step is to create a database of experimental or simulation patterns to feed the network for learning. To this end, 203 different samples in terms of temperature and $\phi$ were prepared for MWCNT–Al_2_O_3_ (30–70%)/oil SAE40 hybrid nanofluid. Usually, the raw data is divided into 70% for training, 15% for validation, and 15% for testing. The validation data prevents the ANN from overtraining using Early stop and generalizes the ANN results. In this study, 203 samples were gained from 7 different $\phi$, 6 various temperatures, and 5 shear rates. The experimental data used for training the ANN is shown in Table 1.

**Table 1:** $\mu_{nf}$ versus temperature with different $\phi$.

T (°C)	$\phi$ (%)						
	1	0.75	0.5	0.25	0.125	0.0625	0
**(a) Shear rate value 100 RPM**							
25	444	407	369.4	347.8	311.2	293.4	343.1
30	315	292	268	255	229	216	249
35	231	216	197	189	164.1	154.7	180.9
40	167.8	157.5	146.2	136.9	122.8	115.3	130.6
45	128.4	120	110.6	105	95.6	86.9	99.4
50	100.3	94.7	85	80.6	71.9	68.1	76.3
**(b) Shear rate value 200 RPM**							
25	434.1	401.2	375	342.5	306.9	288.8	338.1
30	308.4	285.9	263.4	249.4	225	210.9	244.7
35	225	209.1	193.1	183.7	163.1	152.5	177.5
40	166.9	155.6	142.5	135	121.9	114.4	129.8
45	126.3	118.1	108.7	103.1	92.5	85.8	98.4
50	98.8	92.5	83.9	78.8	71.3	66.6	75.9
**(c) Shear rate value 300 RPM**							
25	430.6	394.4	363.8	336.6	302.3	284.1	332.8
30	305	281.9	260.6	246.2	221.9	207.5	241.9
35	222.5	206.3	190	180.6	160.8	150.9	175.3
40	164.1	152.3	140.6	133.1	120	113	128.3
45	164.1	152.3	140.6	133.1	120	113	128.3
50	96.6	90.9	82.9	77.6	70.5	66	75
**(d) Shear rate value 400 RPM**							
30	302.3	278.9	257.8	242.8	218.4	205.3	239.5
35	219.8	203.9	188.4	178.6	159.4	149.2	173.3
40	162.8	151.1	139.5	132	118.9	111.8	127.2
45	123.8	115.1	106.9	100.5	90.7	84.1	96.3
50	96.4	90	81.9	77.2	69.7	65	74.4
**(e) Shear rate value 500 RPM**							
30	298.9	276	255	240	216	202.5	237
35	217.5	202.1	187.5	176.3	157.2	147.5	171.6
40	161.2	149.7	138.8	130.3	117.8	110.3	126.2
45	122.5	114.4	105.6	99.1	90	83	95.4
50	94.7	88.8	81.2	76.1	68.8	64.3	73.7

**Figure 2:**
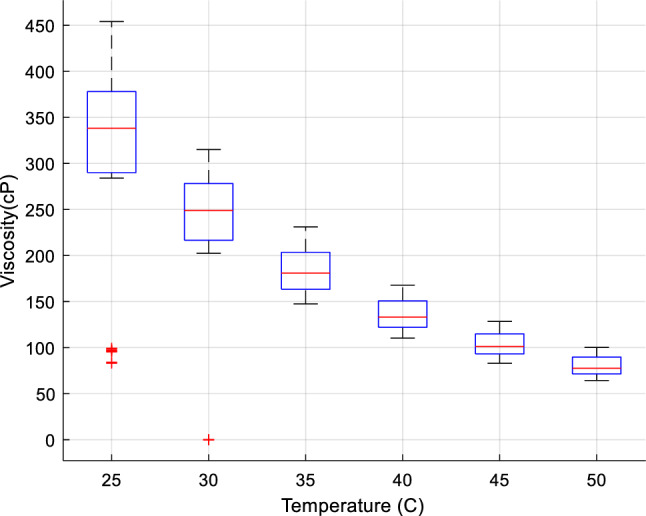
$\mu_{nf}$ deviation based on temperature values.

**Figure 3:**
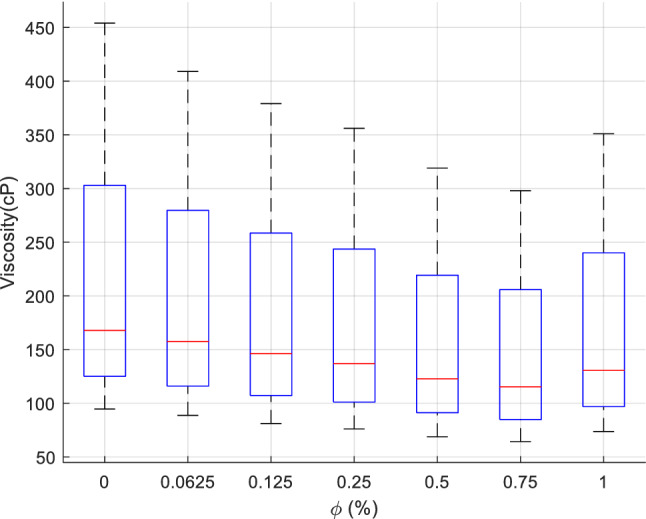
$$\mu_{nf}$$ variation versus $$\phi$$.

**Figure 4:**
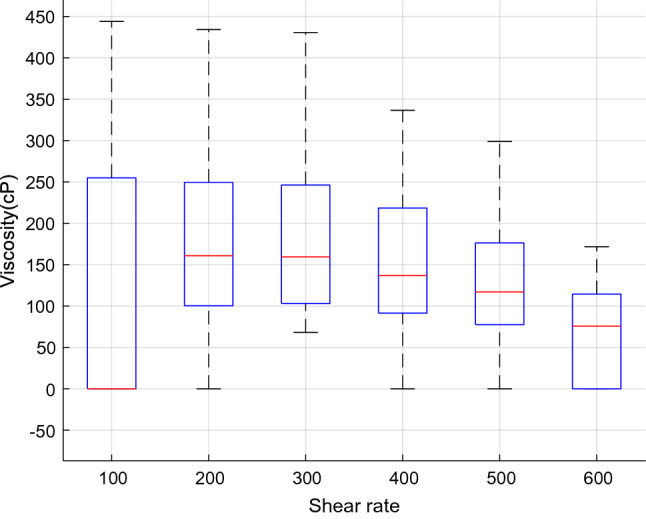
The $\mu_{nf}$ variation versus shear rate.

The deviation of $\mu_{nf}$ and its range is presented in Fig. 2 based on different temperatures, in Fig. 3 versus $\phi$, and Fig. 4 based on various shear rates.

The first step in using ANN is to train the ANN with 70% of the training data and assess the ANN training status; then, the 15% of data that is not used for training is fed, and their performance is computed using the comparison between true and true values and real ANN output. The ANN with the highest performance (the lowest error level) is chosen as the best ANN for the simulation of the system. Due to the random nature of training weights of the ANN, each ANN is trained 50 times from random with each training method, and the model with the highest performance is chosen as a model for the system. As indicated previously, determining the number of neurons in the hidden layer has an important influence on the modeling performance; hence various topologies (number of neurons in hidden layer and transfer function) were considered for each ANN, and the best combination is determined trial and error. The best performance is obtained using 10 neurons with tangent-hyperbolic sigmoid function in the second and linear transfer output layer. Detailed information on ANN configuration is shown in Table 2.

**Table 2:** The ANN topology.

Network parameter	Information
ANN morphology	(Multi-layer perceptron)
Network type	(Feed forward)
Training method	(BackPropagation)
Error criteria	(MeanSquareError(MSE))
Best training method	Trainbr
Number of hidden layers	l Layer
Hidden layer function	tansig, logsig
Output layer function	Purelin
Number of training data	142
Number of validation data	30–31
Number of test data	30–31

## Training methods

**Figure 5:**
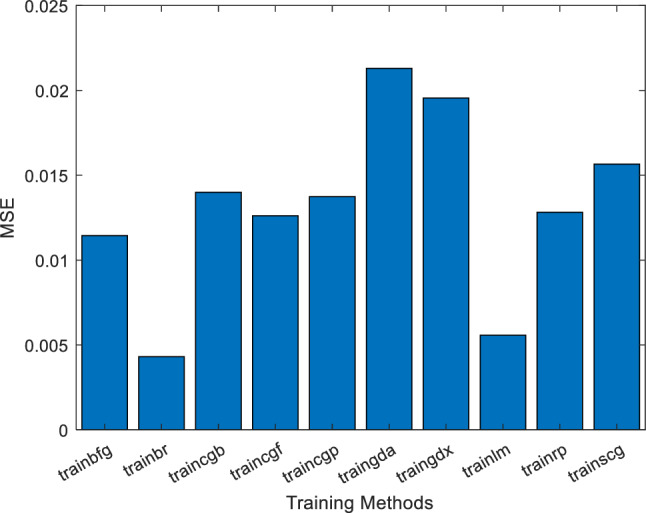
A comparison of error value for different training methods in MATLAB.

One novelty of this study is to obtain the more effective method for ANN training which is more robust and has the highest performance in *μ*_*nf*_ estimation. For this reason, several training methods for feed-forward perceptron ANNs have been used in MATLAB software (Stable release: R2021a/March 17, 2021)^63–68^. The error value for each method alongside other information (including function name in MATLAB and the method e explanation) are gathered in Table 3. Moreover, a detailed comparison of different training methods' error rates is presented in Fig. 5.

**Table 3:** Error value comparison of various training methods in MATLAB software.

MSE	Acronym	Algorithm	Description
1.14E−02	BFG	trainbfg	BFGS quasi-Newton
4.31E−03	BR	trainbr	Bayesian regularization back propagation
1.40E−02	CGB	traincgb	Conjugate gradient with Powell/Beale restarts
1.26E−02	CGF	traincgf	Fletcher–Powell conjugate gradient
1.37E−02	CGP	traincgp	Polak–Ribière conjugate gradient
2.13E−02	GDA	traingda	Gradient descent with adaptive learning rate backpropagation
1.95E−02	GDX	traingdx	Variable learning rate back propagation
1.28E−02	RP	trainrp	Resilient back propagation
1.57E−02	SCG	trainscg	Scaled conjugate gradient
5.58E−03	LM	trainlm	Levenberg–Marquardt

The presented mean square error (MSE) between the actual output and target values for the various forward-based training methods. Other methods have also been reviewed, which have been omitted due to inconsistencies with the physics of the problem and non-convergence of the answers. Among the applied training methods, the trainbr method showed to be the best training method for this problem due to the highest performance; therefore, this method will be explained and analyzed in the form of Regression and Performance graphs.

## Trained ANN performance

**Figure 6:**
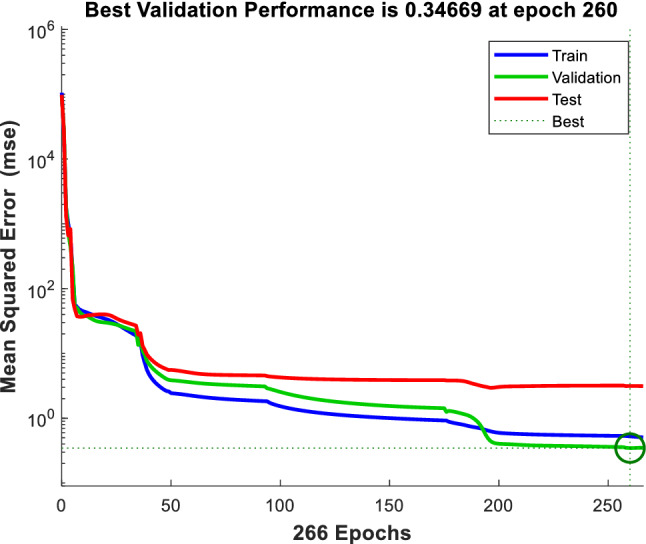
Performance diagram of *μ*_*nf*_ output.

One of the most important indexes showing the training state of an ANN is the Performance graph, which presents the variation of MSE versus training stages. The performance chart of $\mu_{nf}$ is viewed in Fig. 6 for MWCNT–Al_2_O_3_ (30–70%)/oil SAE40 nanofluid. The MSE in the vertical axis is presented in this chart versus training iteration on the horizontal axis (Epoch). Three different data, including Training, Validation, and Test, are observed in this figure representing MSE for the trained points, validation, and test points, respectively. At the first training stage, that ANN has random weights, the MSE value is at the highest level, while after several iterations of training, it is reduced. In this figure, the MSE value for training data is much lower than the rest (validation and test) at the stop iteration. This is the effect of the Early stop strategy for preventing overlearning and displays that the untrained new points have higher MSE rates than trained points fed to the system. The best stop time for the highest performance is indicated with a green circle in the figure, with the lowest MSE in the total iterations. As there are 50 different ANNs for each training scheme, the ANN with the least MSE is the best solution to estimate the *μ*_*nf*_ for any combination of inputs.

**Figure 7:**
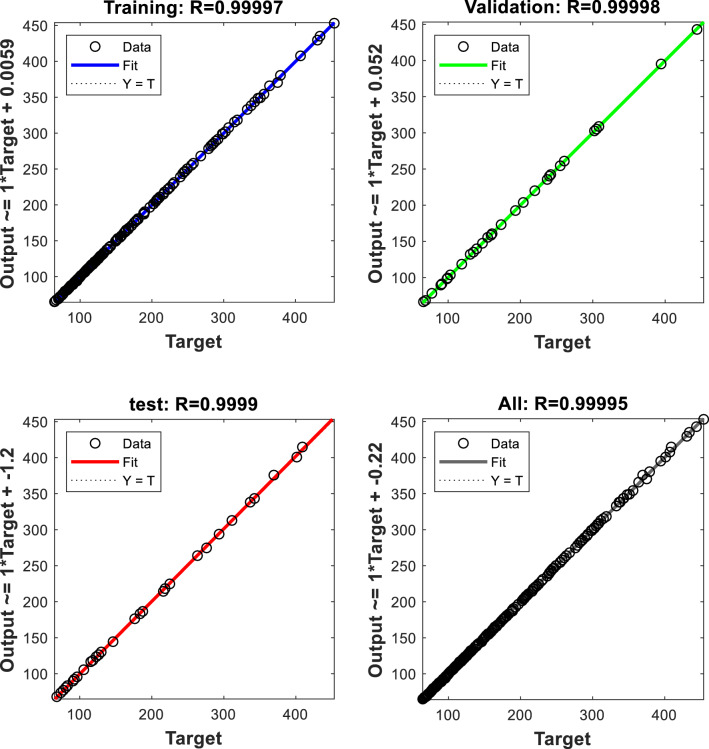
Regression diagram for $\mu_{nf}$ output.

Another indicator to determine the ANN training state is the Regression diagram and the correlation factor between the actual output data and the target values. This diagram is presented in Fig. 7 for $\mu_{nf}$ and shows the correspondence of real ANN outputs and desired values. The horizontal axis corresponds to target values in this graph, while the vertical axis shows the ANN output. In this figure, three different parameters are important. These indexes include correlation coefficient value (R), slope value (M), and bias (B). An ideal ANN must have the same output as target values, and in this condition, the correlation value and slope are equal to 1, and the bias value should be equal to 0. It can be seen that in all four graphs, the slope of the regression line is almost equal to 1; hence it can be deduced that the results are output values of the ANN that have satisfactory accuracy and are sufficiently close to target values. Moreover, the scattering style of points is at a minimum level, and all of the points are located on the bisector of the plane.

In Table 4, the error rate of trained ANNs is presented for $\mu_{nf}$, and it is noticeable that the error value is very low and has an acceptable margin.

**Table 4:** Trained ANNs error rates for $\mu_{nf}$.

System output	The lowest percentage of error	The highest percentage of error	MSE
$\mu_{nf}$	− 1.4	1	4.3e−3

**Figure 8:**
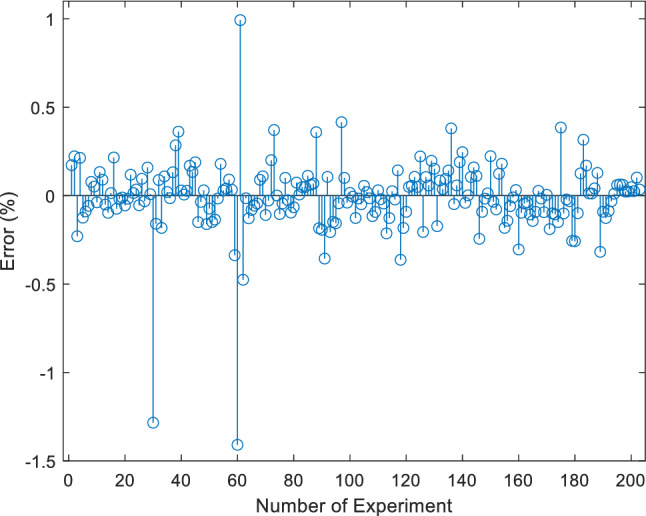
Experimental data error value for $\mu_{nf}$.

The error diagram of different experimental data of $\mu_{nf}$ is presented in Fig. 8. It indicates that the frequency of zero-range error values is very high, proving that the ANN is well trained and creates a good estimation for $\mu_{nf}$.

**Figure 9:**
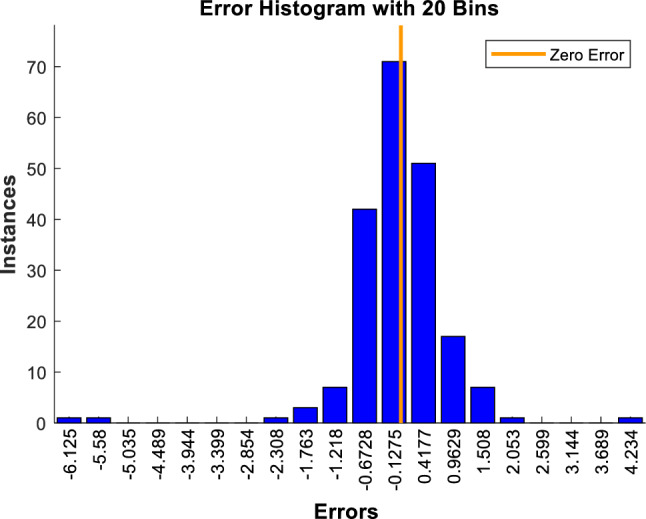
The ANN error histograms for $\mu_{nf}$.

Another important clue for a well-trained ANN is a histogram error value, as shown in Fig. 9. The histogram chart depicts the frequency or count of errors in different error margins ​​in a bar chart. Therefore, the frequency of errors in the vertical axis is presented versus different error margin values in the horizontal axis. The more near-zero frequencies, the more accurate ANN. In this figure, the Zero Error line is indicated by red color. It is seen that most bins with a high frequency of errors are gathered around this line, which is affirmative proof of a suitable choice of training method and its acceptable outcome.

**Figure 10:**
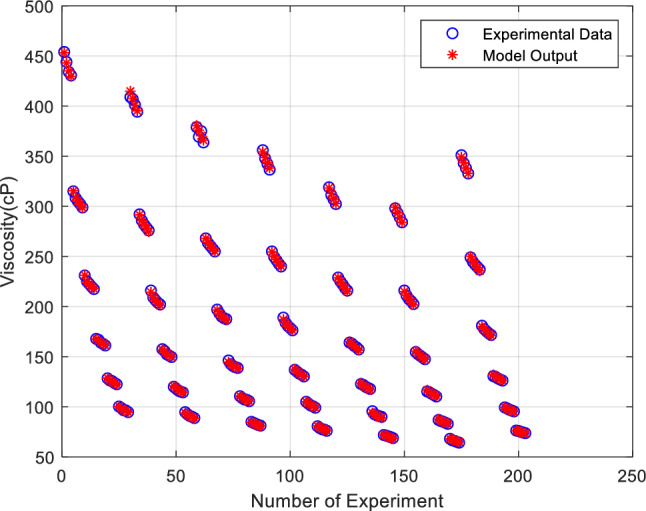
Comparison of ANN outputs and target values for $\mu_{nf}$.

Another comparison between actual data and ANN outputs is presented in Fig. 10 for $\mu_{nf}$. As shown in Fig. 10, a very good agreement between these data can be seen, which shows proper ANN training.

## Analyzing untrained data

**Figure 11:**
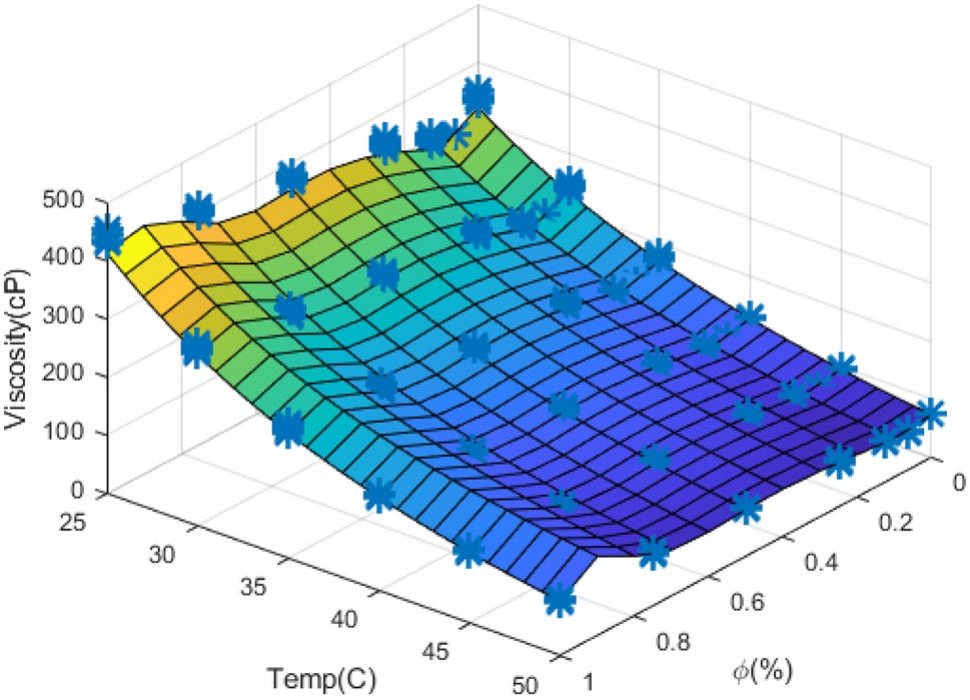
3D graph of $$\mu_{nf}$$.

Another comparison chart is presented in 3D space for $\mu_{nf}$ versus temperature and $\phi$ to investigate the trained ANN's appropriateness for predicting $\mu_{nf}$ for untrained data (see Fig. 11).

This graph shows the ANN output for untrained points in colored mesh, while the trained points are depicted with red stars. Thus, we can see that the stars are located on the 3D surface, and a close correspondence exists between trained ANN results for untrained data and the target values. Based on all of the diagrams presented for the trained ANNs, we can conclude that a well-trained ANN can be used as an approximating function for predicting $\mu_{nf}$. In addition, the $\phi$ has a more significant influence on $\mu_{nf}$ value in contrast to a temperature which has a negligible effect on the output values.

## Conclusion

In summary, the dynamic viscosity of MWCNT–Al_2_O_3_/oil SAE40 nanofluid is investigated at different nanoparticle percentages and temperatures by ANN. In this study, the best topology of the ANN was determined by trial and error, and a two-layer with 10 neurons in the hidden layer with the tansig function had the best performance. Also, to analyze the effect of various training algorithms on the performance of $\mu_{nf}$ prediction, 10 different training functions were used. The results show that a well-trained ANN is created using the trainbr algorithm and showed an MSE value of 4.3e−3 along 0.999 as a correlation coefficient for predicting $\mu_{nf}$. To parameters of nanoparticle percentages and temperatures have a significant effect on the dynamic viscosity. Therefore, an increase $\phi$ has an impressive growth of $\mu_{nf}$ value for all temperatures. And by increasing the temperature, the $\mu_{nf}$ will decrease for all various $\phi$. At the same time, this decrement is more noticeable in higher $\phi$. For example, an increase of the temperature from 25 to 50 °C changes the *μ*_*nf*_ of the pure fluid by only about 200%, while the same changes of temperature in $\phi$ = 1% will cause a 350% drop in *μ*_*nf*_. The academic community and industrial society can use the obtained data in the present manuscript to find the optimal condition in the preparation and production of nanofluids to reduce the energy consumption of industrial instruments. As a suggestion, we recommend using other configuration of neural networks including GMDH network and comparing the obtained results in this paper with the ones obtained through other methods. Moreover, implementation of experiments with other parameters or the same parameters used in this manuscript with other margins is highly recommended for better understanding of this hybrid nano-fluid.
